# Molecular dynamics simulations explore effects of electric field orientations on spike proteins of SARS-CoV-2 virions

**DOI:** 10.1038/s41598-022-17009-1

**Published:** 2022-07-29

**Authors:** Zhifeng Kuang, John Luginsland, Robert J. Thomas, Patrick B. Dennis, Nancy Kelley-Loughnane, William P. Roach, Rajesh R. Naik

**Affiliations:** 1 Materials and Manufacturing Directorate, Air Force Research Laboratory, WPAFB, Dayton, OH 45433 USA; 2 Work Performed With Confluent Sciences, LLC, Albuquerque, NM 87111 USA; 3 711th Human Performance Wing, Air Force Research Laboratory, JBSA Fort Sam Houston, San Antonio, TX 78234 USA; 4 Air Force Office of Scientific Research, Arlington, VA 22203 USA; 5 711Th Human Performance Wing, Air Force Research Laboratory, WPAFB, Dayton, OH 45433 USA

**Keywords:** Viral infection, Biological physics

## Abstract

Emergence of the severe acute respiratory syndrome coronavirus 2 (SARS-CoV-2) and its current worldwide spread have caused a pandemic of acute respiratory disease COVID-19. The virus can result in mild to severe, and even to fatal respiratory illness in humans, threatening human health and public safety. The spike (S) protein on the surface of viral membrane is responsible for viral entry into host cells. The discovery of methods to inactivate the entry of SARS-CoV-2 through disruption of the S protein binding to its cognate receptor on the host cell is an active research area. To explore other prevention strategies against the quick spread of the virus and its mutants, non-equilibrium molecular dynamics simulations have been employed to explore the possibility of manipulating the structure–activity of the SARS-CoV-2 spike glycoprotein by applying electric fields (EFs) in both the protein axial directions and in the direction perpendicular to the protein axis. We have found out the application of EFs perpendicular to the protein axis is most effective in denaturing the HR2 domain which plays critical role in viral-host membrane fusion. This finding suggests that varying irradiation angles may be an important consideration in developing EF based non-invasive technologies to inactivate the virus.

## Introduction

**Figure 1:**
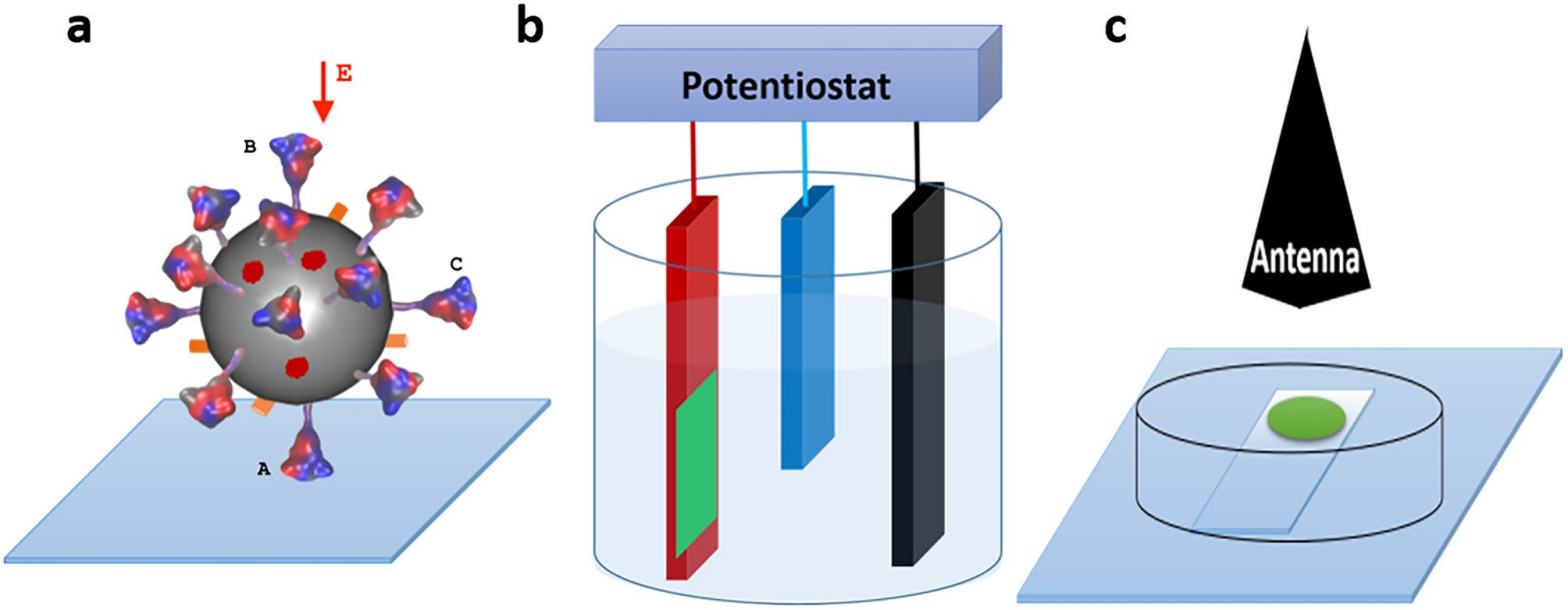
The relevant experiments. (**a**) Schematic drawing of a virion adsorbed on surface. A spike protein at position A, B and C differently senses the applied external field E when the virion is attached on surface. At position A, the protein sees the direction of E aligning with its axis. At position B, the protein sees the direction of E antiparallel to its axis. At position C, the protein sees the direction of E perpendicular to its axis. (**b**) Schematic drawing of virions (green) adsorbed on a working electrode (red). A static external electric field can be maintained between the working electrode and the reference electrode (blue). The black bar represents the counter electrode. (**c**) Schematic drawing of a horn antenna illuminating virions (green) adsorbed on a glass slide. The image is designed and drawn by Z.K. for illustration.

The Coronavirus Disease 2019 (COVID-19) pandemic has posed an extraordinary threat to human health and public safety. The pandemic is caused by human-to-human transmission of the severe acute respiratory syndrome coronavirus 2 (SARS-CoV-2). The viral infection may cause mild to severe, and even fatal respiratory illness^1–3^. The SARS-CoV-2 virion is a pleomorphic particle encapsulated by a lipidic envelope. Among the proteins anchored in the envelope, the spike (S) proteins extending radially from the envelope are responsible for binding of the virus to host cell receptors and host-viral membrane fusion to initiate infection. Figure 1a shows a schematic drawing of a virion on surface.

Vaccination is currently the most effective measure against SARS-CoV-2 virus. Yet, the effectiveness is limited by the rapid viral mutation and vaccine life cycle. The manufacture, transport and storage of vaccine are costly. To develop other prevention strategies, researchers are developing not only vaccines and drugs, but also non-invasive physical therapies and decontamination approaches for detecting and destroying the virus in public spaces^4–6^. It is especially interesting to identify technologies to neutralize virions indiscriminate of variants and to disinfect surfaces contaminated with the virus. Electric fields have been used in food processing to inactivate microorganisms and enzymes that compromise food safety and shelf life^7^. After adsorption of human immunodeficiency virus type 1 (HIV-1) onto a working electrode (Fig. 1b), the application of a constant potential 1.0 V for 3 min was carried out using a potentiostat. It has been reported that about 90% infection of (HIV-1) was inhibited^8^. It has been demonstrated that the illumination of microwaves (Fig. 1c) at a certain frequency can achieve 100% inactivation ratio of influenza A virus H3N2^9^. Based on clinical evidence, an electrical stimulation method has been proposed to inhibit SARS-CoV-2 reproduction^10^. Most recently, it is reported that irradiating the S1 subunit of the S protein with a 700 W, 2.45 GHz electromagnetic field (EMF) for 2 min can denature the S1 subunit to around 95%, while the temperature is controlled at 37 °C^11^. To effectively implement and optimize EF effects, a better understanding of the actual mechanisms for the inactivation is required. The parameters such as the microwave intensity, frequency, exposure time, power density and pulse repetition frequency to gain the most effective inactivation need to be determined. Determination of physical forces acting on the virion will enable more precise tuning of those parameters.

It is well understood that electric fields (EF) cause rotation of dipoles and motion of charged particles, which increase the system’s kinetic energy and thus its temperature, and also result in friction between the molecules to generate heat. The changes due to local heating are known as thermal effects^12,13^. While thermal heating is used to kill coronavirus, temperatures higher than 60 °C are required, resulting in protein denaturation^14,15^. To raise sample temperatures higher than 60 °C, excess of 100 W microwave power at 2.45 GHz is required, which is beyond the IEEE C95 safety standards for permissible exposure to humans^16^. For the development of non-invasive technologies, the non-thermal effect of electric fields has been explored and reported in the literature^7,17^. Such non-thermal effect can refer to for example, the EF-induced structural rearrangement of membrane lipids and protein conformation without local heating^18^. It is reported that non-thermal effects of microwaves are able to inactivate influenza A virus H3N2 by physically rupturing the viral envelope^9^.

While distinguishing thermal and non-thermal effects of electric fields on structure–activity of macro biomolecules is rather complicated in an experimental setting^7^, Molecular dynamics (MD) simulation has advantages of controlling parameters and factors. MD simulations with external electric fields have been used to study a wide variety of aqueous, nanoscale and biological systems such as electroporation of membrane and modulation of protein structures^19,20^. For example, the effects of electric and low-frequency microwave fields ranging from 0.1 to 1.5 V/nm on hen egg white lysozyme were examined using nonequilibrium MD simulations. Changes in the protein’s secondary structure relative to the zero-field state was observed above 0.1 V/nm^21^. A series of MD simulations for bovine insulin monomer were carried out up to 1 $\upmu$s in the presence of EF with strengths ranging from 0.15 to 0.60 V/nm, respectively. The results showed that the secondary structure of insulin remained intact under the external electric field strength below 0.15 V/nm^22^. Conformational changes of a short alanine-based peptide exposed under EFs ranging from 0.1 to 0.5 V/nm were investigated. It was shown that a minimum field strength of 0.1 V/nm was required to perturb the zero-field behavior of the peptide^23^.

To explore a non-invasive therapeutic option for treating Alzheimer’s disease by applying electric fields, molecular level understanding of EF effects on Amyloid-$\beta$ aggregation has attracted great effort in MD simulation community. MD simulations were carried out to study the influence of a constant electric field of 20 mV/nm on the conformations of the membrane-embedded amyloid-$\beta$(29–42) peptide dimer. It was shown that conformational changes were induced by EF^24^. Effects of static EF ranging from 0.2 to 0.5 V/nm on amyloid-$\beta$ aggregation were also studied, where it was shown that the application of EFs significantly perturbed the conformational structure of a well-ordered protein^25^.

Most recently, non-equilibrium MD simulations were performed to study the potential effects of EF on the structure of a truncated receptor binding domain (RBD) fragment (residues 319–686) in the chain A from prefusion model 6VSB (PDB ID) of the SARS-CoV-2 S protein, where the RBD is in an open form. It was reported that severe damage of the fragment structure occurs under weak EF ${10}^{-5}$ V/nm^26^. This work has shown the possibility of simulating experimentally comparable EF intensity. Here we are interested in the following questions:

1. It is commonly accepted that each monomer of the S protein is composed of two subunits S1 and S2, and the N-terminal domain (NTD) and RBD in the S1 subunit collaboratively function^2^. Are the results from the truncated RBD applicable to the entire S protein with glycans?
2. The time scale of the above-mentioned vitro experiments^9,11^ for the protein denature is in minutes. Current MD simulations are not able to directly access the experimental timescales^27^. An alternative approach widely used in the above-reviewed literatures is to use high EFs (0.1–0.5 V/nm) for submicrosecond simulations to gain insights into the EF effects. But the MD simulation performed by Arbeitman et al. took only 700 ns to denature the protein under a low EF 10^−5^ V/nm. Is the discrepancy due to the facts that the open-form of the truncated RBD is in a metastable state and an electric field is more effective to denature a much smaller protein?
3. When a virion is adsorbed on solid surfaces as schematically shown in Fig. 1, the RBDs are most likely in closed forms^2^. The mobility of the virion on surfaces is limited and each S protein takes a certain orientation^28^. When an external electric field is applied, each S protein senses that field differently. As schematically shown in Fig. 1a, the S protein at the position A experiences the EF in a direction parallel to its axis. At the position B, the S protein experiences the EF in a direction anti-parallel to its axis. At the position C, the S protein experiences the EF in a direction perpendicular to its axis. How does the S protein with RBDs in closed forms respond to the applied EF at different locations on the envelope?

In this paper, we will investigate how the full S protein with RBDs in closed forms responds to the applied external electric field at representative positions A, B and C of a virion immobilized on solid surfaces, as shown in Fig. 1a. The non-equilibrium molecular dynamics study of impacts of EF directions on proteins can be traced back to the study of aquaporin gating mechanism^29,30^.

## Results

### The spike glycoprotein model

The S protein is a homo-trimeric complex. Each monomer has two subunits (S1 and S2), which are composed of 1273 residues decorated with 22 *N*-glycans and 1 *O*-glycan^31^. To compare with previous studies^26^ and experimental data^32^, we further divide each monomer into eight domains: the signal peptide and N-terminal domain (S1-NTD: 1–318), the receptor binding domain (RBD: 319–686), the S2 N-terminal domain (S2-NTD: 687–797), the fusion peptide to the central helix domain (FP-CH: 798–1016), the $\beta$-hairpin and subdomain 3 (BHSD3: 1017–1104), the linker region (L: 1105–1144), the heptad repeats 2 domain (HR2: 1145–1178), and the S2 C-terminal domain (S2-CTD: 1179–1273).

**Figure 2:**
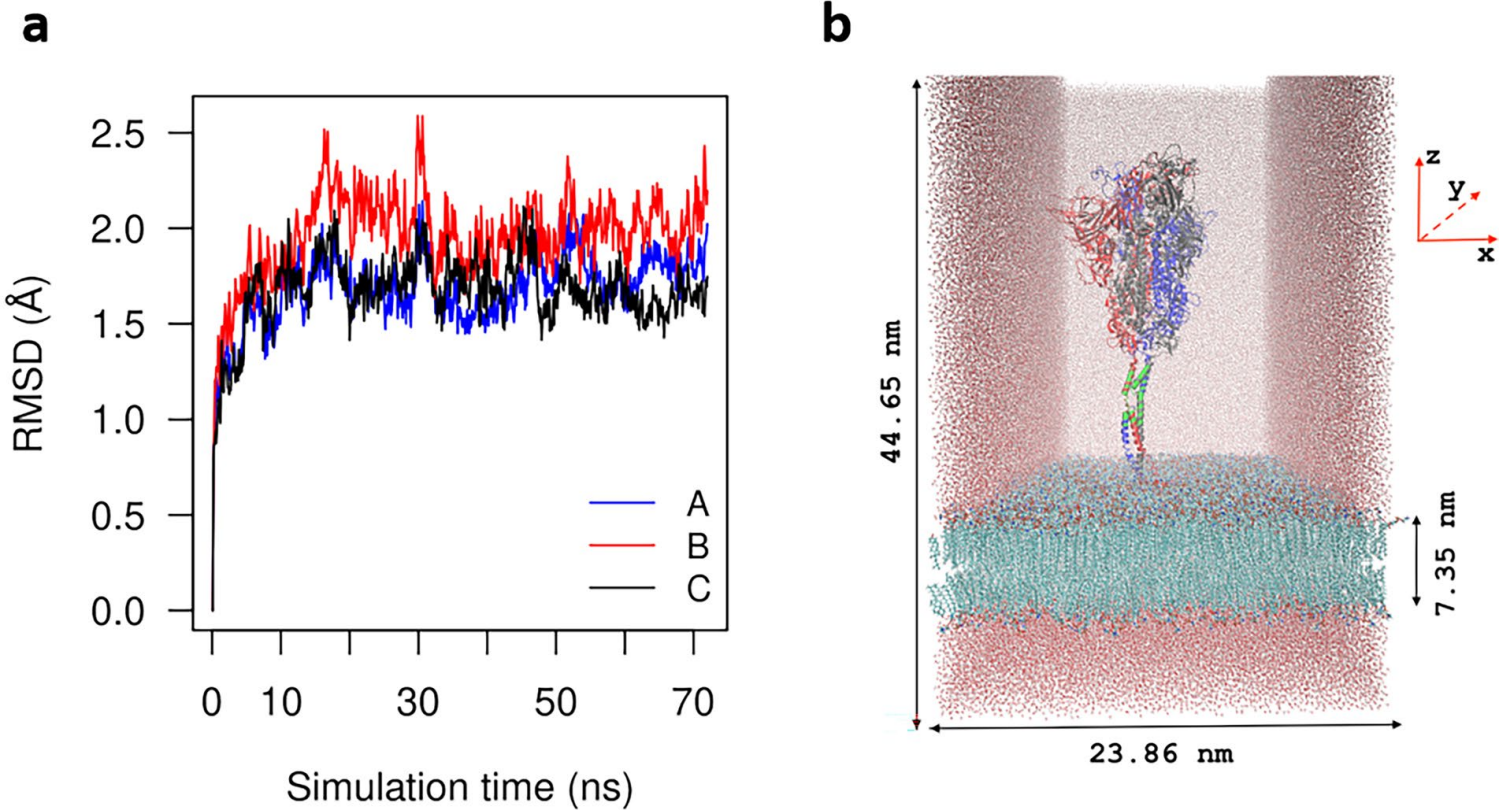
The simulation model. (**a**) The root-mean-square deviations (RMSD) from its restart snapshot in the last 70 ns show that equilibrium has reached. (**b**) The end-point configuration of the equilibrated system. Protein is inserted in a viral membrane. The green stalk is the HR2 domain. For clarity, partial water and ions are shown.

Membrane fusion between viruses and host cells requires S protein to be cleaved into S1 and S2 subunits by furin. In the uncleaved materials such as solid surfaces, a majority (83%) of the spike glycoprotein is in a closed conformation^33^. So we have chosen the published fully glycosylated full-length closed conformation model 6VXX_1_1_1 in a viral membrane as the initial model for MD simulations^31^. The protein and membrane are solvated in a rectangular TIP3P water box neutralized with 0.15 M KCL solution. The total number of atoms in the system is 2,260,694. The system is equilibrated for 2.5 $\upmu\mathrm{s}$ in an isothermal isobaric ensemble (1 atm, 308.15 K). The root-mean-square deviation (RMSD) from its restart snapshot in the last 70 ns is shown in Fig. 2a. In the last 30 ns, the RMSD remains at $1.8\pm 0.2 \;$Å, indicating that equilibrium has achieved. The dimension of the equilibrated unit cell is $23.86 \; \mathrm{nm}\times 23.56 \; \mathrm{nm}\times 44.65 \; \mathrm{nm}$, centered at the origin. The membrane is located between $-18.644 \; \mathrm{nm}$ and $-11.296 \; \mathrm{nm}$ (min and max of the z-coordinates of all membrane atoms) normal to Z direction, giving a thickness $7.35 \; \mathrm{nm}$, which is close to cryo-EM estimation $7.8\pm 0.7$ nm of the envelope thickness of the coronavirus prototype murine hepatitis virus (MHV)^1^. The snapshot of the end-point configuration is shown in Fig. 2b. The overall shape is in consistent with the image obtained from tomographic data^34^. The ectodomain head (1–1136) is supported by a long stalk containing three hinges marked by hip (1137–1141), knee (1159–1173), and ankle (1194–1212). The hip links the head and the upper leg (1142–1158). The knee links the upper leg and the lower leg (1174–1193). The ankle links the lower leg and foot (1213–1273).

### Effects on protein secondary structures

On average, 24–40 copies of S proteins are randomly distributed on the envelope surface of a viral particle^34,35^. When a virion is attached on a surface as shown in Fig. 1, its rotation ability is limited. When an external EF is applied, the S proteins at different locations may sense the same applied EF differently so that some may be affected and other may not be affected. At the position A shown in Fig. 1a, the force is parallel to its axis. At B, the force is anti-parallel to its axis. At C, the force is perpendicular to the protein axis. Based on previous MD studies reviewed in the introduction section, we have chosen a high field strength 0.2 V/nm to investigate which part is most sensitive to EF in S protein in a reasonable simulation time. Non-equilibrium MD simulations (NEMD) in the presence of EF 0.2 V/nm have been performed in three directions: parallel to S protein axis (Z+), anti-parallel to S protein axis (Z−) and perpendicular to S protein axis (X+) to resemble S proteins at locations A, B, and C in Fig. 1a.

**Figure 3:**
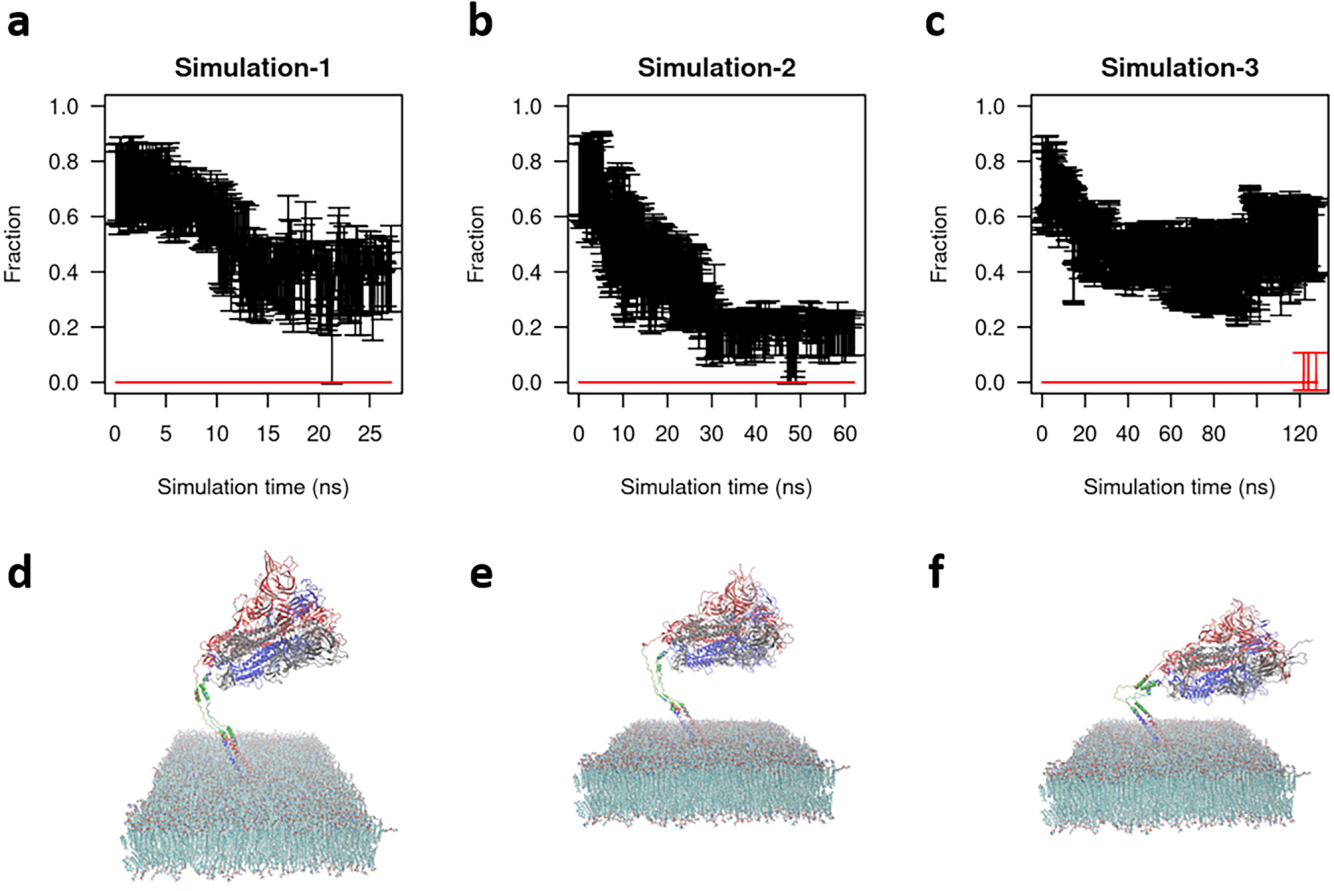
The denaturation of HR2 domain. (**a**–**c**) Three independent simulations show that the fraction of alpha helix significantly decreases from 0.72 ± 0.12 to 0.38 ± 0.16. Black curves represent fraction of alpha helix content with error bars. Red lines represent fraction of beta sheet content. (**d**–**f**) The corresponding end-point configuration of the protein-membrane.

When the ratio of the simulation unit cell in the x–y plane is kept constant while allowing fluctuations along all axes, membrane electroporation has been observed within $1 \; \mathrm{ns}$ simulation time in an isothermal isobaric ensemble (1 atm, 308.15 K). This is in agreement with previous studies^36^. A representative snapshot of the pore is shown in Supplementary Fig. S1. Since our objective is to investigate the impacts of EF directions on S protein, we have carried out three independent simulations in the NVT ensemble with membrane atoms fixed. The secondary structure contents have been calculated using the STRIDE (STRuctural IDEntification) algorithm^37^ implemented in visual molecular dynamics (VMD)^38^. It is seen from Fig. 3a–c that all three simulations show that the fraction of alpha helix in HR2 domain significantly decreases from $0.72\pm 0.12$ to $0.38\pm 0.16$. The corresponding end-point configuration of the protein-membrane are shown in Fig. 3d–f. It is clearly seen that the HR2 domain (green stalk in Fig. 3d–f) becomes coiled. The protein head bows down toward the membrane. From Supplementary Fig. S2–10, it is interesting to see that except for HR2 when EF is applied in X+ direction, no significant secondary structure changes have been observed in other domains in all three simulations.

### Effect on protein charge distribution

**Figure 4:**
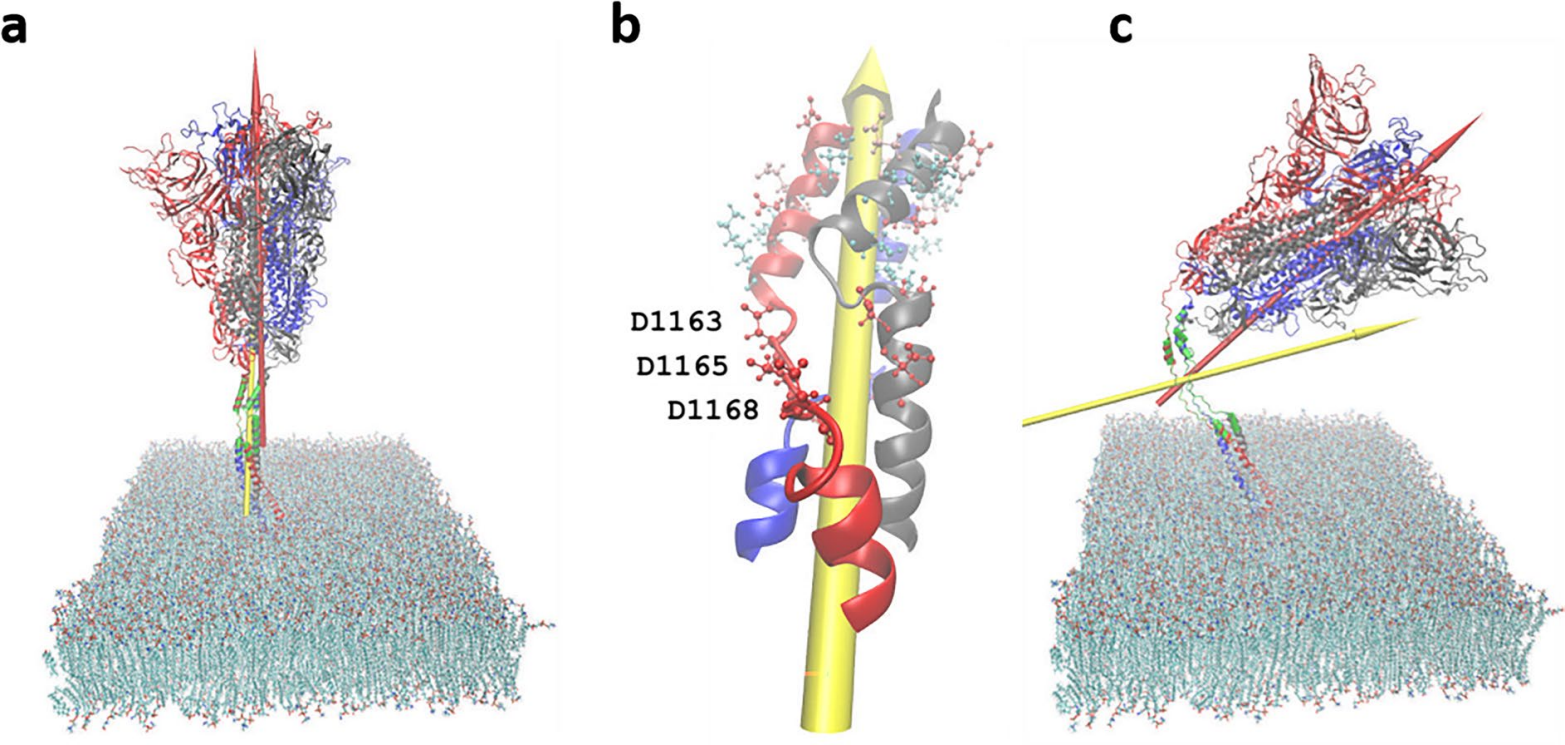
The driving force for the denaturation of HR2. (**a**) The spike protein dipole moment perpendicular to membrane (red arrow). When EF are applied in the direction perpendicular to membrane, torque is very small. (**b**) The enlarged HR2 segment. Red CPK represent Aspartic acids. Pink CPK represent Glutamic acid. Cyan CPK represent Lysine. When EF are applied in the direction X+ perpendicular to the protein dipole, a torque acts on the HR2 dipole (yellow arrow: dipole moment of HR2). A force pulls D1163–D1168 to the left so that protein bends at D1163–D1168. (**c**) the end-point conformation after 0.2 V/nm applied in X+ direction for 25 ns in simulation-1. The length of arrows is not proportionally scaled.

The direct interaction between EFs and protein surface charges may cause side chains of a residue to rotate. These subtle changes may be captured by calculating the protein dipole moments. Table 1 shows the protein dipole moments in three independent simulations. In the first column named zeroEF, the dipole moments have been calculated using equilibrated conformation when there is no applied EFs. The S protein possesses a large intrinsic dipole. The amplitude of the dipole moment is $23313.34\pm 30.22$ Debye. The direction approximately aligns with Z axis, since the angle between the dipole moment and the Z axis is $6.50\pm 1.47$ degree. Figure 4a shows the dipole moment within the S protein. In the columns named X+, Z+ and Z−, the dipole moments have been calculated using the end-point snapshots when 0.2 V/nm EFs are applied in the direction perpendicular, parallel and anti-parallel to the protein axis. In average, the amplitude changes in dipole moment between Z+, Z− and zeroEF are only 100.05 and − 113.33 Debye, respectively. The angles almost remain the same. But the amplitude changes between X+ and zeroEF is as large as 1721.11 Debye. The angles in X and Z directions have significant changes from 88.78° to 31.60 in X, from 6.50 to 58.76 in Z. The significant changes in dipole moment indicate a large conformational change when EFs are applied in X+ direction.

**Table 1:** Change of S protein dipole moments in response to EF 0.2 V/nm in three independent simulations S-1, S-2 and S-3. Comparing with zero EF applied, no significant changes observed in Z+ and Z− directions. But there are significant changes in amplitude and angles when EF is applied in X+ direction. A is the amplitude in unit Debye. θx, θy, and θz are the angles in unit degree between dipole moment and X, Y, Z axis, respectively.

	Zero EF				X+				Z+				Z−			
	A	θx	θy	θz	A	θx	θy	θz	A	θx	θy	θz	A	θx	θy	θz
S-1	23,347.3	90.1	84.5	5.5	23,745.7	43.4	84.8	47.1	23,537.1	90.9	82.8	7.2	23,258.6	91.5	84.5	5.7
S-2	23,289.3	86.1	82.8	8.2	24,206.1	30.5	85.4	59.9	23,978.4	86.0	78.8	11.9	23,358.4	86.7	77.0	13.4
S-3	23,303.5	90.2	84.1	5.9	27,151.5	20.9	87.3	69.3	22,724.7	87.5	83.0	7.4	22,983.1	88.6	87.2	3.1
AVE	23,313.3	88.8	83.8	6.5	25,034.5	31.6	85.8	58.8	23,413.4	88.1	81.5	8.9	23,200.0	88.9	82.9	7.4
STD	30.2	2.3	0.9	1.5	1847.8	11.3	1.3	11.2	636.0	2.5	2.4	2.7	194.4	2.5	5.3	5.4

We have also calculated the dipole moments of the short HR2 bundle without EF applied, with EF applied in X+, Z+ and Z− directions. Table 2 shows noticeable difference with and without EF applied can only be observed when EF is applied in X+ direction. Therefore, all these calculations show that the most effective EF direction to impact the S protein structure is X+.

**Table 2:** Change of HR2 domain dipole moments in response to EF 0.2 V/nm in three independent simulations S-1, S-2 and S-3. Comparing with zero EF applied, no significant changes observed in Z+ and Z− directions, but there are significant changes in amplitude and angles when EF is applied in X+ direction. A is the amplitude in unit Debye. θx, θy, and θz are the angles in unit degree between dipole moment and X, Y, Z axis, respectively.

	Zero EF				X+				Z+				Z−			
	A	θx	θy	θz	A	θx	θy	θz	A	θx	θy	θz	A	θx	θy	θz
S-1	229.5	88.9	57.1	33.0	546.6	15.9	87.8	74.3	333.3	88.2	48.9	41.2	262.7	94.3	55.8	34.6
S-2	261.5	87.6	30.7	59.5	766.6	8.7	89.5	81.3	284.3	82.7	47.0	43.9	346.6	89.8	44.4	45.6
S-3	255.5	87.5	61.2	28.9	712.4	14.2	94.6	103.4	237.1	77.5	35.2	57.7	257.4	83.3	54.1	36.7
AVE	248.9	88.0	49.6	40.4	675.2	12.9	90.6	86.3	284.9	82.8	43.7	47.6	288.9	89.2	51.4	38.9
STD	17.0	0.8	16.6	16.6	114.6	3.8	3.6	15.2	48.1	5.3	7.4	8.9	50.0	5.5	6.1	5.8

### The driving force for denaturation

The secondary structure changes represent protein backbone conformation changes. Since the temperature is maintained as a constant during the MD simulations, the observed denaturation of HR2 must be caused by the direct interactions between EFs and protein charges. Indeed, the net charge of the whole protein with 3819 residues is − 21 e. But the short bundle with only 102 residues has a net charge − 12 e. The charged residues in HR2 domain in each monomer are: E1150, E1151, D1146, D1153, D1163, D1165, D1168, K1149, K1154, K1157. Electric force applied in X+ direction pulls the bundle towards the X− direction at the position of D1163–D1168 as shown in Fig. 4b so that the protein head quickly (within 120 ns) bows down towards membrane as shown in Fig. 4c. Meanwhile, torque on the dipole untwists the helix. Another reason that HR2 is the most vulnerable part exposed to EF attack is because the electric field is more effective at denaturing a smaller protein. HR2 domain is a small three helix bundle like a stalk whereas other domains pack together like a big head.

## Discussion

Previous thermal stability studies have shown that the spike protein in a closed conformation is very stable^15,33,39^. Temperature controlled microwave irradiation has shown that 2 min are required to denature around 95% of the S1 subunit of S protein at 37 °C under a high-power 700 W, 2.45 GHz electromagnetic field^11^. Here, using non-equilibrium molecular dynamics simulations, we have predicted that the most vulnerable part to electric field stimulus is the HR2 domain in the S2 subunit of S protein. A high degree of denaturation of the HR2 domain can be made when EFs are applied in the X+ directions.

**Figure 5:**
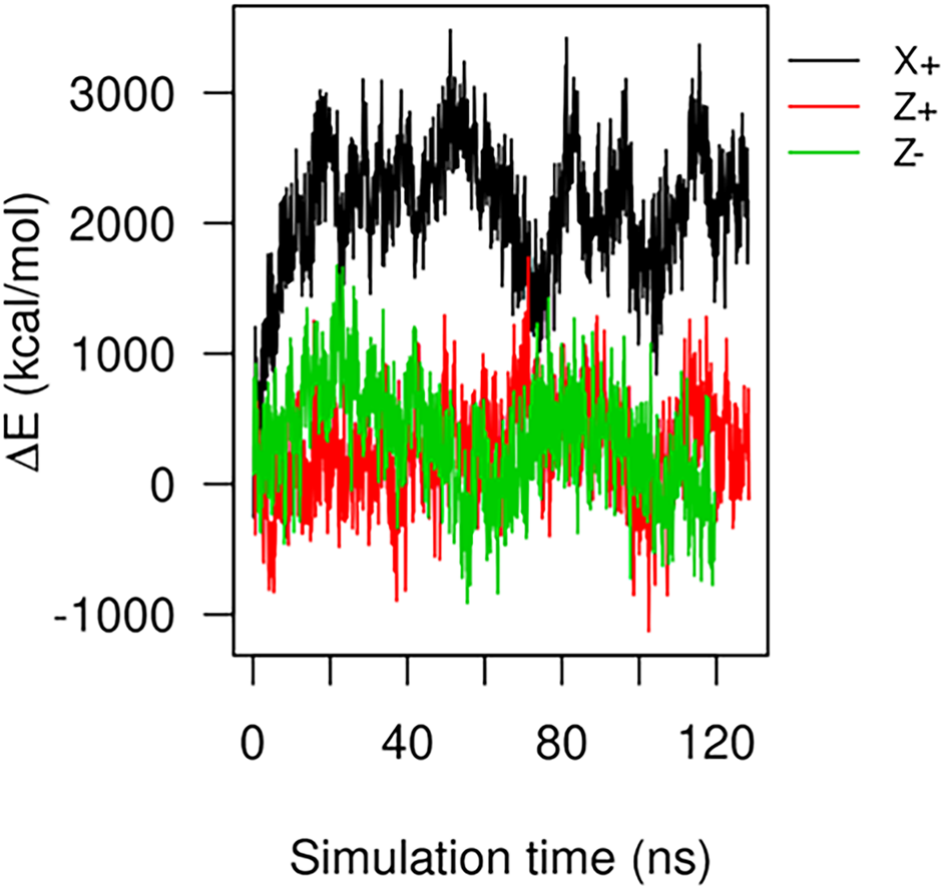
The evolution of the potential energy of the spike protein during ~ 120 ns nonequilibrium MD simulation. The potential energy of the equilibrated S protein before application of external force is set as the zero-reference base line. While 0.2 V/nm EF is applied in Z+ and Z− directions, the potential energy fluctuates around zero, which indicates no significant charge rearrangement. While 0.2 V/nm EF is applied in X+ direction, the potential energy deviates from its equilibrium value, which indicates significant charge rearrangement. An energy barrier appears at 51.1 ns. The bumpy energy profile after 50 ns indicates that the process is irreversible after the removal of EFs.

To investigate whether the damage is reversible after the removal of external EFs, we have shown the potential energy evolution of the S protein along the recorded trajectories during ~ 120 ns simulations in Fig. 5. It is seen that the potential energies fluctuate around its equilibrium value when EFs are applied in Z+ and Z− directions, which indicates no significant charge rearrangements. However, when EFs are applied in the X+ directions, the potential energies significantly deviate from its equilibrium value, which indicates large conformational change. The largest energy barrier appears at 51.1 ns. The bumpy potential energy profile after 50 ns implies that the process is irreversible after the removal of the external force.

HR2 domain plays a critical role in host-viral membrane fusion to enable viral entry to host cells. The correct folding of HR2 is critical for the formation of the central HR1–HR2 six-helix bundle, which brings the viral membrane close to the host membrane for fusion. Since HR2 domain is completely exposed to solution, it has been proposed as a potential accessible target for developing vaccines and therapeutics^32^. Thus it can be reasonably speculated that the denaturation of HR2 results in prohibition of viral entry into the host cell and leads to potential EF-based approaches into disinfecting surfaces contaminated by SARS-CoV-2.

Our ongoing research is to answer the following questions: (1) what is the minimum field strength required to cause noticeable denaturation of the protein in MD simulations? (2) What is the minimum simulation time is required to observe noticeable secondary structure changes of the protein for experimentally comparable field strength?

We have also realized some common challenges in this research field. When external EFs are applied, the resulting net potential may cause positive and negative ions to flow in an opposite direction so that the periodic boundary conditions of the systems are broken. Fortunately, for EFs less than 0.7 V/nm and simulation time scales less than microseconds, bulk ion separation is negligible so that current flow is also negligible^19,40,41^. Therefore, in our studies, particle-mesh Ewald summation with periodic boundary conditions is still valid for computing the intrinsic reaction potential. Then the applied external potentials are superimposed on the reaction potential to generate the total potential energy surface for atomic motions. However, as seen in Fig. 3f, after 120 ns long simulation the protein is distorted and close to the edge of the simulation box due to the applied external force so that the periodicity is broken for another reason. Therefore, the requirement of periodicity and much longer simulation time have excluded our attempt to directly compare with experimental results obtained in a time scale of minutes, where S1 subunit has been studied^11^. We cannot exclude the possibility of denaturing other protein domains when simulation time is extended.

The 6VXX_1_1_1 model is built based on cryogenic electron microscopy (cryo-EM) structure (PDB ID: 6VXX)^42^. In 6VXX, the coordinates of 972 out of 1273 residues are resolved for each monomer at the resolution $2.8 \;$Å. Using 6VXX as a template, a more complete closed cryo-EM structure has been later resolved and deposited in PDB bank (PDB ID 6ZGE)^33^. In 6ZGE, the coordinates of 1098 out of 1273 residues are resolved for each monomer at the resolution $2.6 \;$Å. The comparison of the resolved residues between 6VXX and 6ZGE are shown in Supplementary Fig. S11. The RMSD of alpha carbons of the equilibrated model 6VXX_1_1_1 from the 6ZGE cryo-EM structure is $4.1\pm 0.1 \;$Å, which is shown in Supplementary Fig. S12. The relatively high RMSD is mainly due to two ill-matched segments: residue id 173–185 and residue id 469–488, as shown in Supplementary Fig. S12. These two segments are resolved in 6ZGE but computationally predicted in 6VXX_1_1_1. This observation implies that there is room for further refinement of the 6VXX_1_1_1 model.

## Conclusions

Using non-equilibrium all-atom molecular dynamics simulations, the impacts of external electric field directions on the spike glycoprotein of SARS-CoV-2 virions attached on inanimate surfaces have been investigated. We have found that the most vulnerable part to electric field stimulus is the HR2 domain in the protein. The short HR2 bundle (residue id 1145–1178) has a net charge − 12 e assuming histidine residues are neutral. The strong electrostatic interactions between EFs applied in perpendicular to the protein axis and charged residues cause rapid structure changes on the HR2 domain. Since HR2 plays a critical role in forming six-helix bundle to facilitate the viral-membrane fusion, electric field induced denaturation of HR2 domain may lead to the development of new technologies in disinfecting surfaces contaminated by SARS-CoV-2 and inhibiting SARS-CoV-2 infection of human cells.

## Methods

### The SARS-CoV-2 spike protein model

Using cryo-EM structures (PDB ids: 6VXX and 6VSB) as templates, several models for the fully glycosylated full-length SARS-CoV-2 spike protein in a viral membrane have been built and deposited at CHARMM-GUI COVID-19 archive (https://charmm-gui.org/docs/archive/covid19)^31^.

We have downloaded the first closed state model (6VXX_1_1_1) as our initial model. In the model, the fully glycosylated full-length SARS-CoV-2 spike protein in a viral membrane is solvated in a water box along with a 0.15 M KCl solution. Each monomer was decorated with 22 N-linked and 1 O-linked glycans. Total 45 disulfide bonds were added.

### Equilibrium molecular dynamics simulation

Proteins, lipids, and carbohydrates were modeled using the CHARMM36-(m) force field^43–48^. Water was modeled using the TIP3P water model^49^. The SHAKE algorithm was used to constrain all hydrogen atoms^50^. The protonation states of all histidine residues were chosen to be neutral with the proton positioned on ${N}_{\delta }$ (HSD). Periodic boundary conditions were imposed in all three directions. The long-range electrostatic interactions were calculated by the particle-mesh Ewald method with a mesh size of 1 Å^51,52^. A 12 Å cutoff was used for nonbonded interactions with a smoothing function implemented after 10 Å.

Molecular dynamics simulations were carried out using the NAMD 2.14 software package^53^. First, the system was minimized for 100,000 steps using the conjugate gradient algorithm^54^. Then the system was gradually heated up from 0 K to 298.15 K, increasing 30 K every 10,000 steps. Third, equilibrations were carried out with time step 1 fs in the constant temperature 298.15 K and constant volume for 10 ns. Forth, equilibrations continued in the constant temperature 298.15 K and constant pressure 1 atm for 9.23 ns while constraints were removed. To fasten the equilibration, we continued the equilibration at a higher temperature 308.15 K with a time step 2 fs until the root-mean-square deviations (RMSDs) of alpha carbons fluctuated around a constant. Totally, 2.5 $\upmu$s equilibrations were carried out. In NPT ensemble, the dimension of the unit cell in the x–y plane is kept constant, while fluctuations were allowed along the z axis.

The Langevin piston Nose–Hoover method^55,56^ was used to control the pressure with piston period 200 fs and damping time scale 100 fs. The temperature was controlled at 308.15 K through Langevin dynamics with damping coefficient 5/ps. In the equilibration processes, harmonic constraints of exponent 2 and force constant 10 kcal/mol/Å^2^ were imposed on protein backbone atoms and lipid head atoms, and gradually released.

### Non-equilibrium molecular dynamics simulation

Since the dimensions of the simulation box are many orders of magnitude smaller than wavelengths of practical interest, a uniform external electric field $\mathbf{E}$ is applied directly to each atom carrying a partial charge ${q}_{i}$. Newton’s equation of motion is given by

1 $${m}_{i}{\ddot{\mathbf{r}}}_{i}={\mathbf{f}}_{i}=-{\nabla }_{{\mathbf{r}}_{i}}U+{q}_{i}\mathbf{E},$$

where ${\mathbf{r}}_{i}$ is the position of the $i$th atom with mass ${m}_{i}$ and partial charge ${q}_{i}$. ${\mathbf{f}}_{i}$ is the force due to interactions with all other atoms via the potential $U$ and applied electric field $\mathbf{E}$. The velocity Verlet algorithm was used to solve the Newton equation^57,58^.

A constant electric field 0.2 V/nm was applied to the equilibrated system in positive Z (Z+), negative Z (Z−), and positive X (X+) direction, respectively. Three independent simulations were performed for each direction in the NVT ensemble with membrane atoms fixed.

### Dipole moment

The dipole moment ($\overrightarrow{d}$) is calculated as:

2 $$\overrightarrow{d}=\sum_{i=1}^{N}{q}_{i}{\overrightarrow{r}}_{i}\hspace{0.33em},\hspace{0.33em}\hspace{0.33em}\hspace{0.33em}{\overrightarrow{r}}_{i}={\mathbf{r}}_{i}-\frac{1}{M}\sum_{i=1}^{N}{m}_{i}{\mathbf{r}}_{i},$$

where $N$ is the total number of atoms in selection, ${\mathbf{r}}_{i}$ is the position of the $i$ th atom with mass ${m}_{i}$ and partial charge ${q}_{i}$, $M=\sum_{i=1}^{N}{m}_{i}$ is the total mass, and ${\overrightarrow{r}}_{i}$ is the position vector of the ith atom relative to the center of mass.

### Secondary structure analysis

The STRIDE (STRuctural IDEntification) algorithm^37^ as a plugin in VMD was used for the assignment of the secondary structure elements.

Images were produced using VMD^38^ and R studio^59^.

## Supplementary Information

Supplementary Figures.

## Data Availability

The datasets and computer codes used to generate and/or analyze the results during the current study are available from the corresponding authors on reasonable request.
