# Supplementary material for: Molecular dynamics simulations explore effects of electric field orientations on spike proteins of SARS-CoV-2 virions
Source: Sci Rep. 2022 Jul 29;12:12986. doi: 10.1038/s41598-022-17009-1 (PMC9334739; doi:10.1038/s41598-022-17009-1)
Supplement: Supplementary file 1 — Supplementary Figures. [file 41598_2022_17009_MOESM1_ESM.pdf]

Supplementary Information

Molecular dynamics simulations explore effects of electric field orientations on spike proteins of SARS-CoV-2 virions

Zhifeng Kuang[1*], John Luginsland[2], Robert J. Thomas[3], Patrick B. Dennis[1],

Nancy Kelley-Loughnane[1], William P. Roach[4], and Rajesh R. Naik[5*]

[1] Materials and Manufacturing Directorate, Air Force Research Laboratory, WPAFB, OH 45433, United States
[2] Work performed with Confluent Sciences, LLC, Albuquerque, NM 87111. Now with Air Force Office of Scientific Research, Arlington, VA 22203, United States
[3] 711th Human Performance Wing, Air Force Research Laboratory, JBSA Fort Sam Houston, TX 78234, United States
[4] Air Force Office of Scientific Research, Arlington, VA 22203, United States
[5] 711th Human Performance Wing, Air Force Research Laboratory, WPAFB, OH 45433, United States

[*] Correspondence: Zhifeng Kuang <zig.kuang@gmail.com>

Rajesh R. Naik <bionanomaterials@hotmail.com>

Supplementary Figures

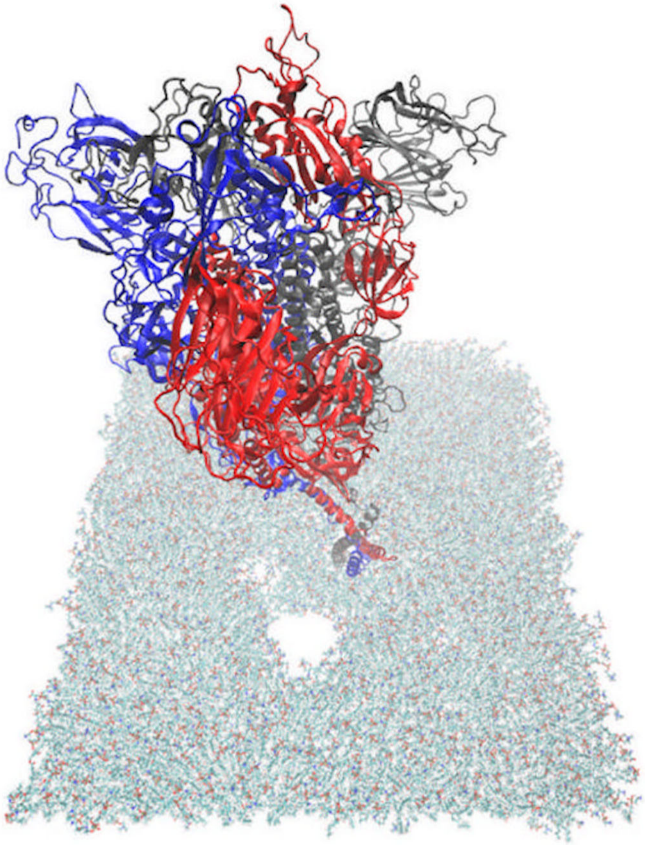

Figure S1. *Pore is observed in membrane within 1ns simulation in an isothermal isobaric ensemble (1 atm, 308.15K) when the ratio of the simulation unit cell in the membrane plane is kept constant while allowing fluctuations along all axes.*

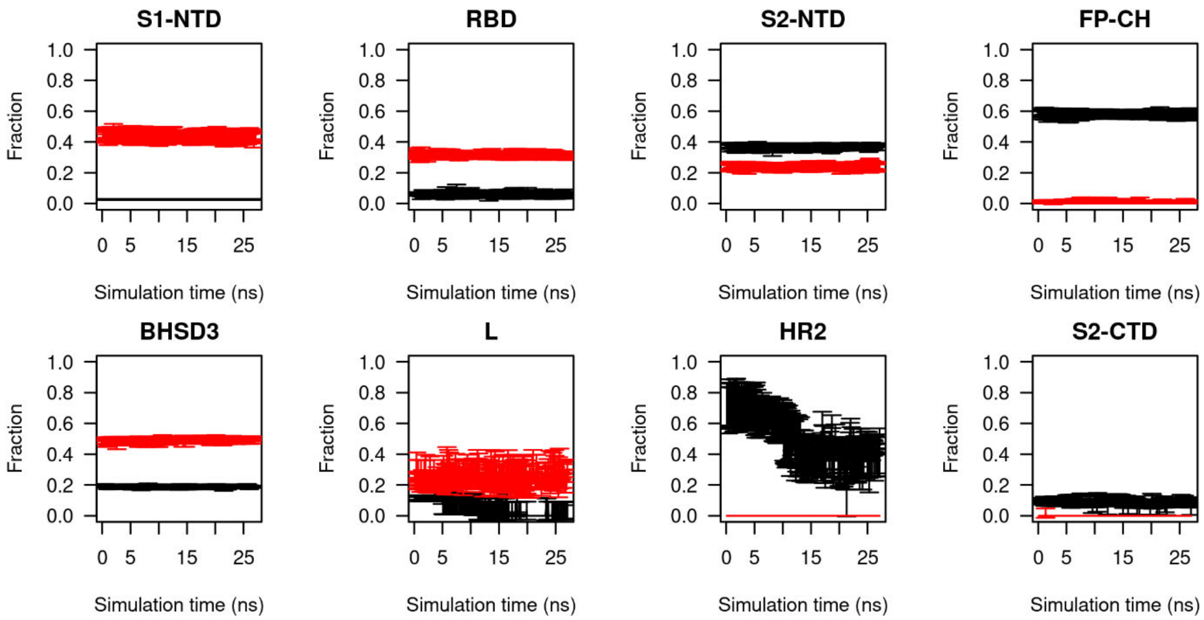

Figure S2. *Secondary structure change of S protein under electric field 0.2V/nm in X+ direction in simulation 1. Time evolution of alpha (black) and beta (red) content of eight sequential segments in non-equilibrium molecular dynamics (NEMD) simulation. Significant secondary structure changes occur in the HR2 domain within 25 ns simulation. Notations are defined in main text.*

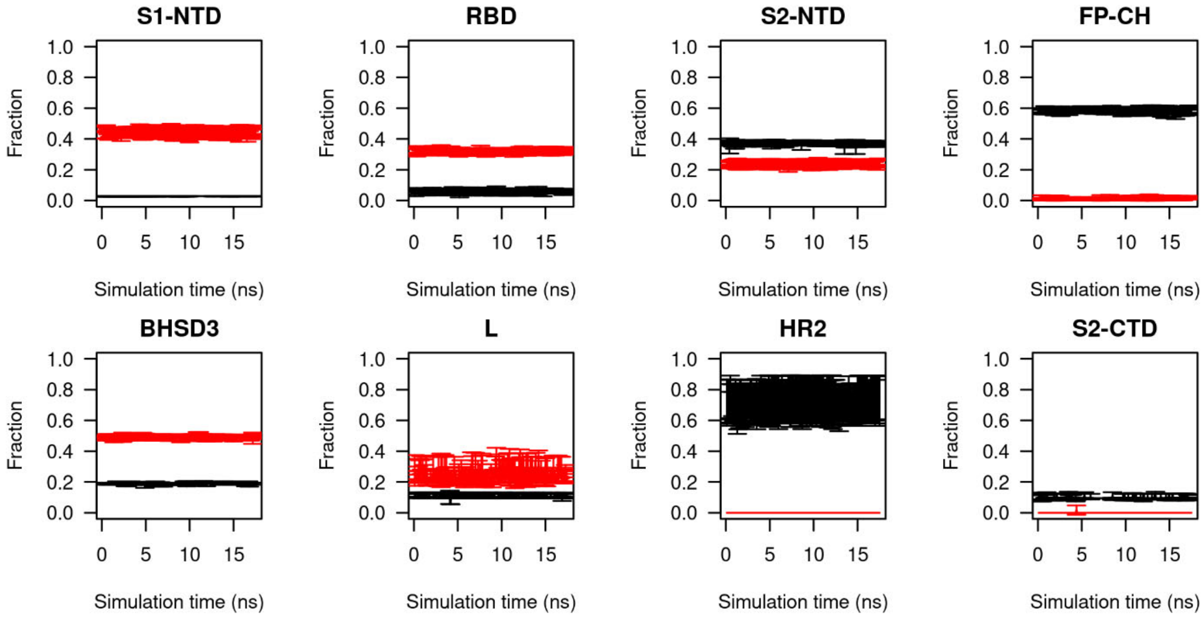

Figure S3. *Secondary structure change of S protein under electric field 0.2V/nm in Z+ direction in simulation 1. Time evolution of alpha (black) and beta (red) content of eight sequential segments in NEMD simulation. No significant secondary structure changes occur within 20 ns simulation. Notations are defined in main text.*

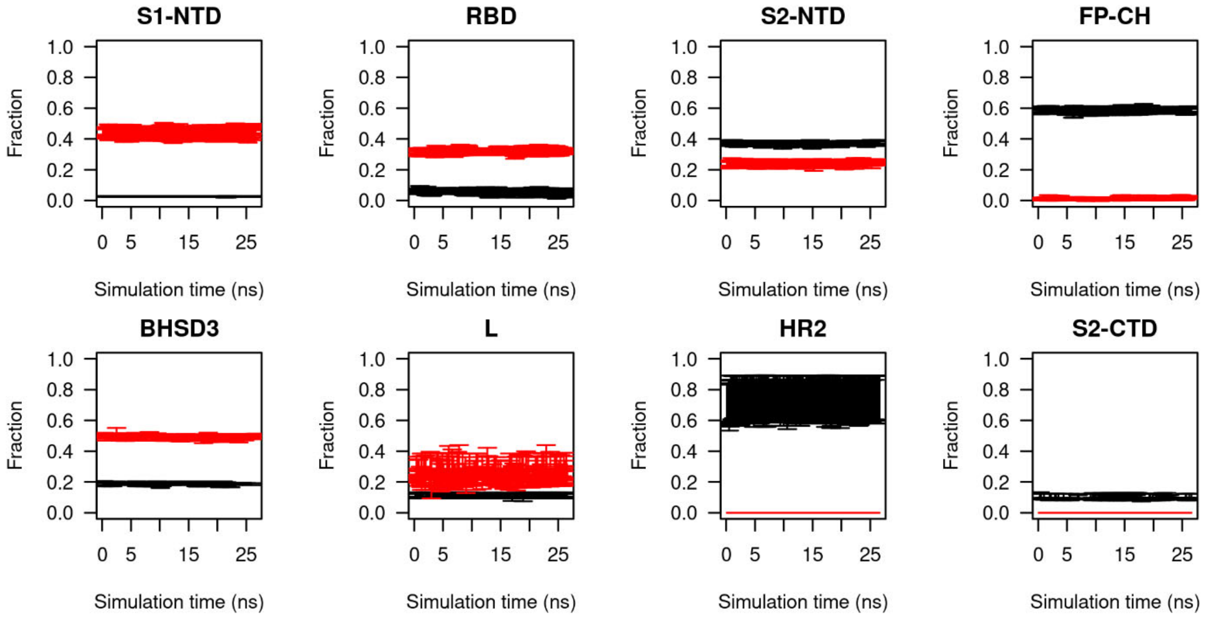

Figure S4. *Secondary structure change of S protein under electric field 0.2V/nm in Z- direction in simulation 1. Time evolution of alpha (black) and beta (red) content of eight sequential segments in NEMD simulation. No significant secondary structure changes occur within 25 ns simulation. Notations are defined in main text.*

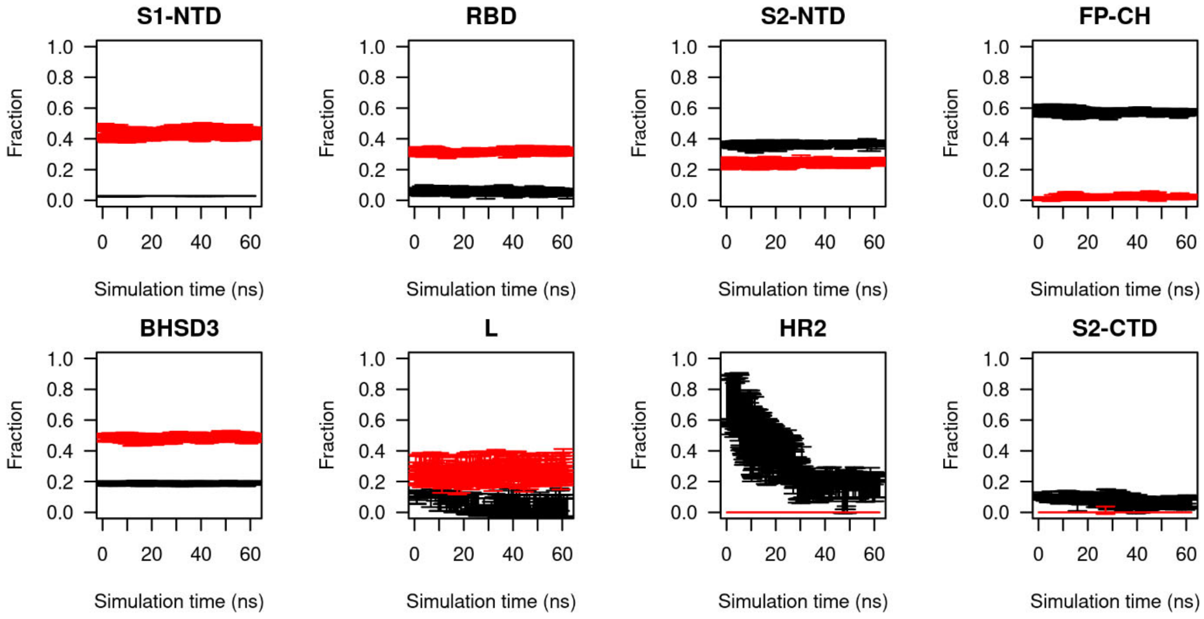

Figure S5. *Secondary structure change of S protein under electric field 0.2V/nm in X+ direction in simulation 2. Time evolution of alpha (black) and beta (red) content of eight sequential segments in non-equilibrium molecular dynamics (NEMD) simulation. Significant secondary structure changes occur in the HR2 domain within 60 ns simulation. Notations are defined in main text.*

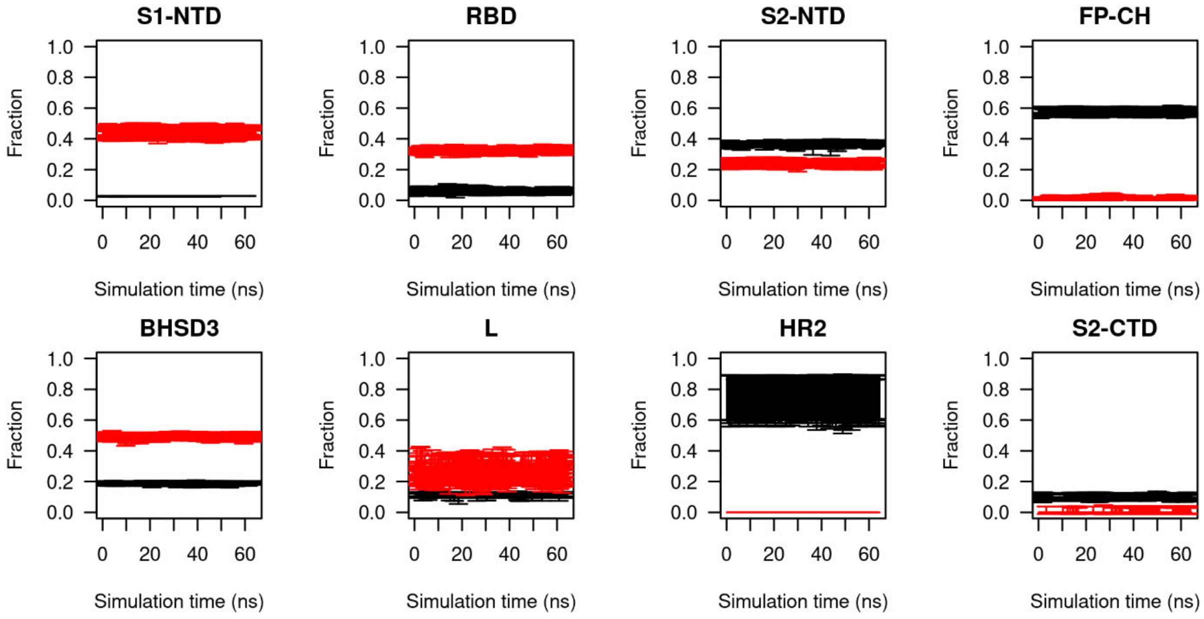

Figure S6. *Secondary structure change of S protein under electric field 0.2V/nm in Z+ direction in simulation 2. Time evolution of alpha (black) and beta (red) content of eight sequential segments in NEMD simulation. No significant secondary structure changes occur within 60 ns simulation. Notations are defined in main text.*

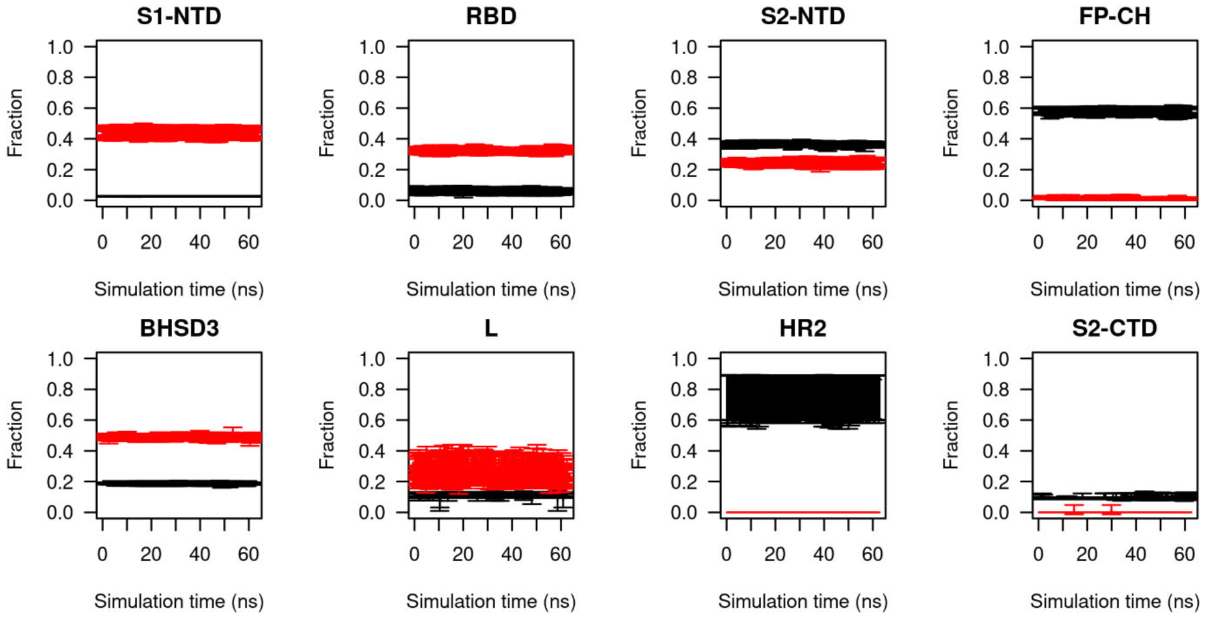

Figure S7. *Secondary structure change of S protein under electric field 0.2V/nm in Z- direction in simulation 2. Time evolution of alpha (black) and beta (red) content of eight sequential segments in NEMD simulation. No significant secondary structure changes occur within 60 ns simulation. Notations are defined in main text.*

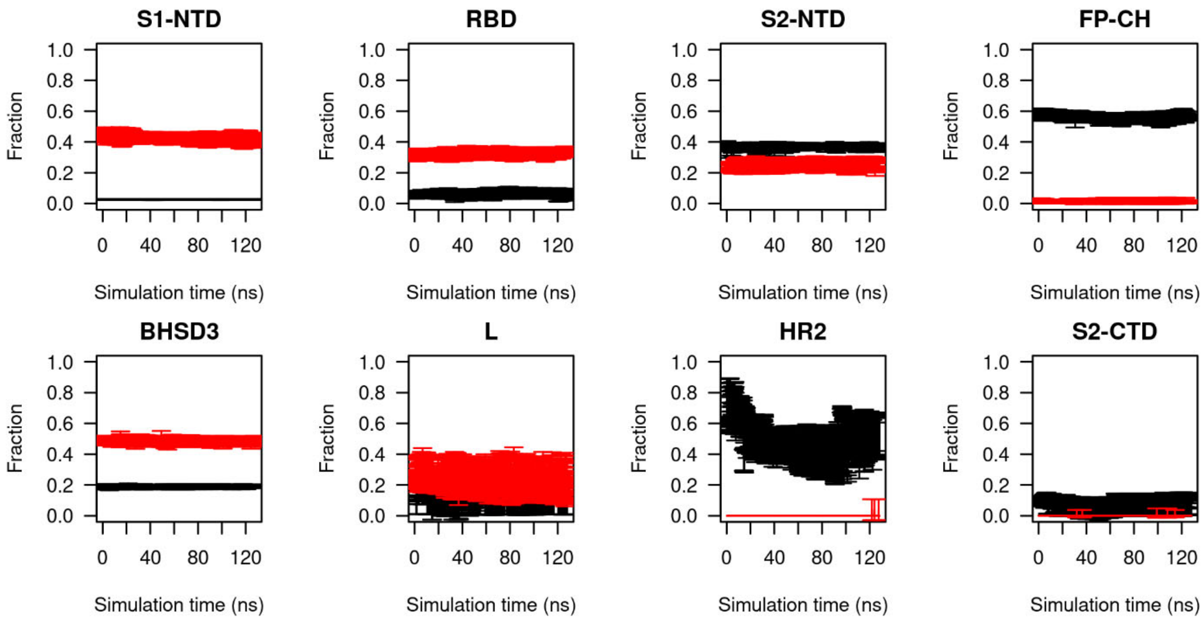

Figure S8. *Secondary structure change of S protein under electric field 0.2V/nm in X+ direction in simulation 3. Time evolution of alpha (black) and beta (red) content of eight sequential segments in non-equilibrium molecular dynamics (NEMD) simulation. Significant secondary structure changes occur in the HR2 domain within 120 ns simulation. Notations are defined in main text.*

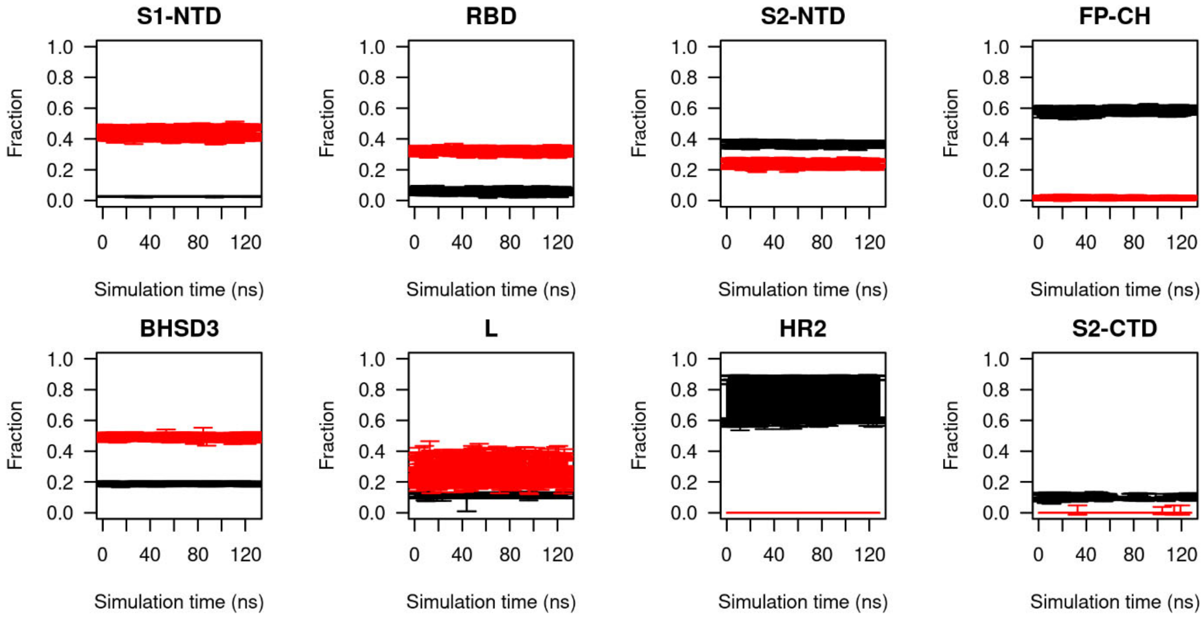

Figure S9. *Secondary structure change of S protein under electric field 0.2V/nm in Z+ direction* in simulation 3*. Time evolution of alpha (black) and beta (red) content of eight sequential segments in NEMD simulation. No significant secondary structure changes occur within 120 ns simulation. Notations are defined in main text.*

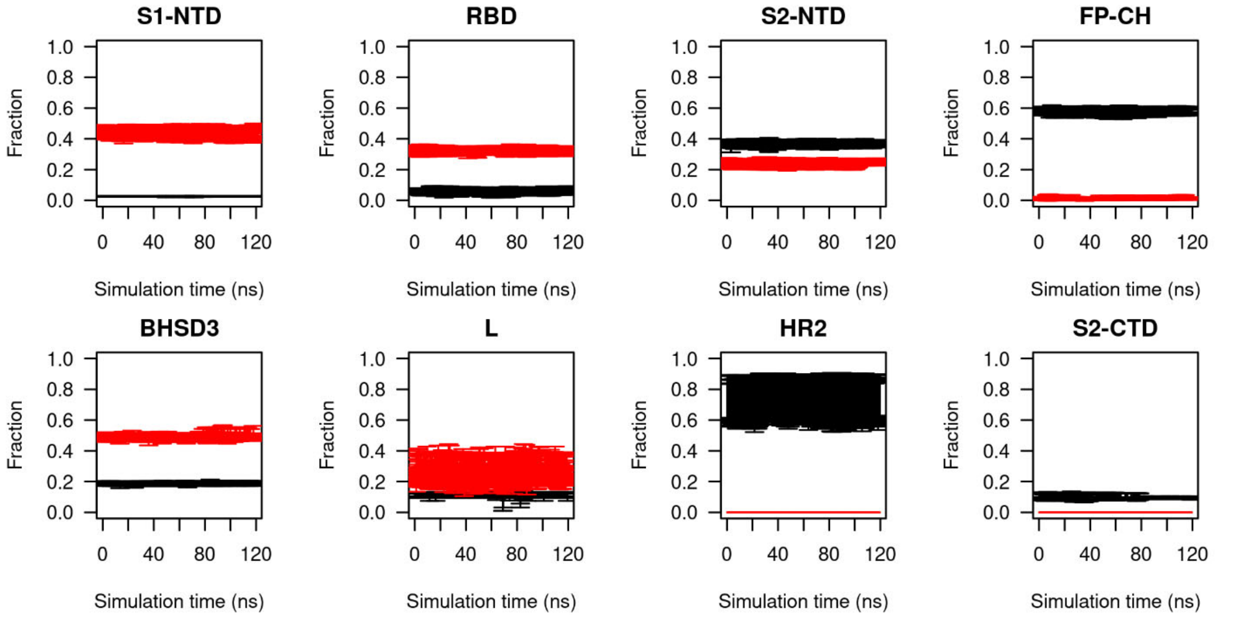

Figure S10. *Secondary structure change of S protein under electric field 0.2V/nm in Z- direction* in simulation 3*. Time evolution of alpha (black) and beta (red) content of eight sequential segments in NEMD simulation. No significant secondary structure changes occur within 120 ns simulation. Notations are defined in main text.*

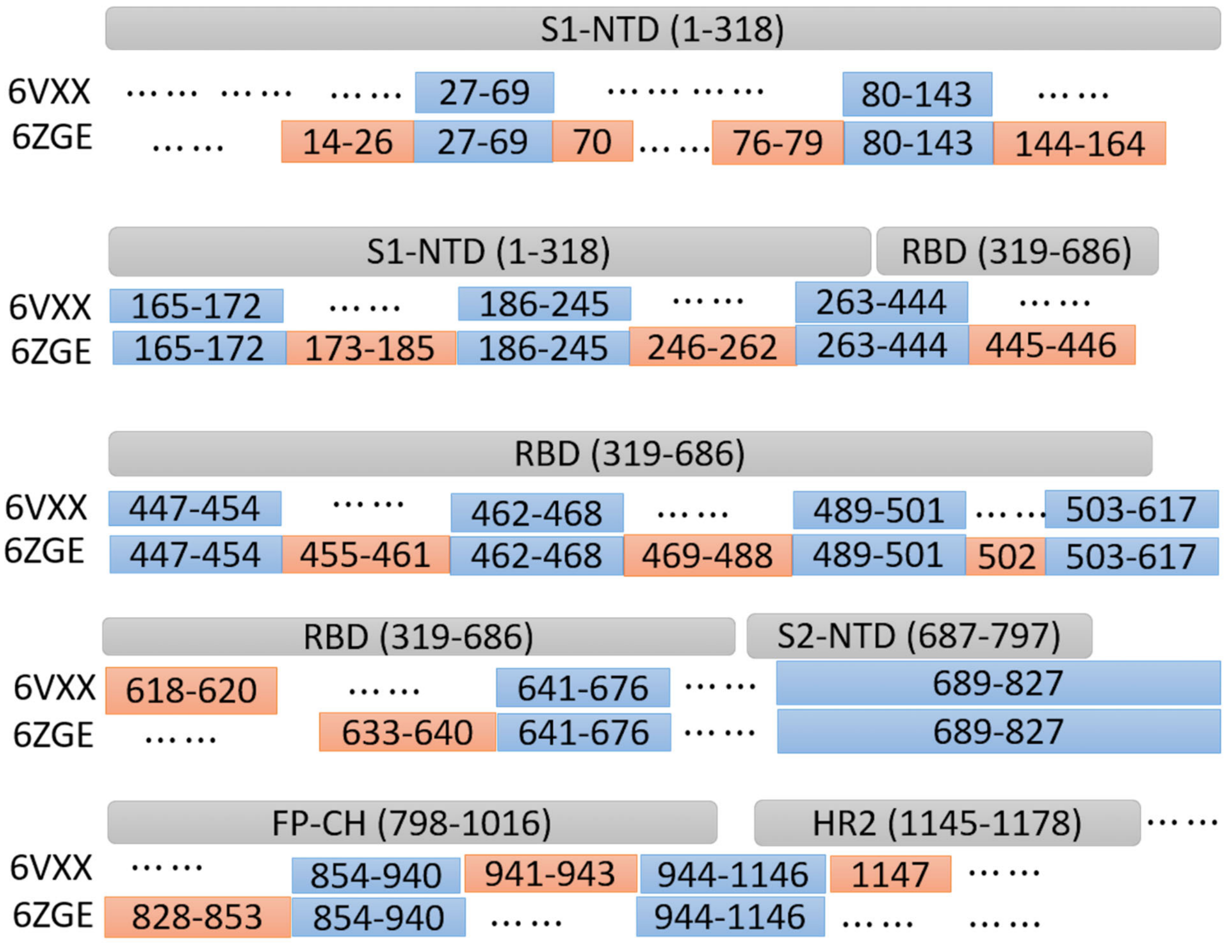

Figure S11. *The residues whose coordinates are determined by Cryo-EM. 6VXX and 6ZGE are PDB IDs deposited in protein data bank. Blue blocks denote those residues appearing in both 6VXX and 6ZGE whereas red blocks denote those residues appearing in either 6VXX or 6ZGE.*

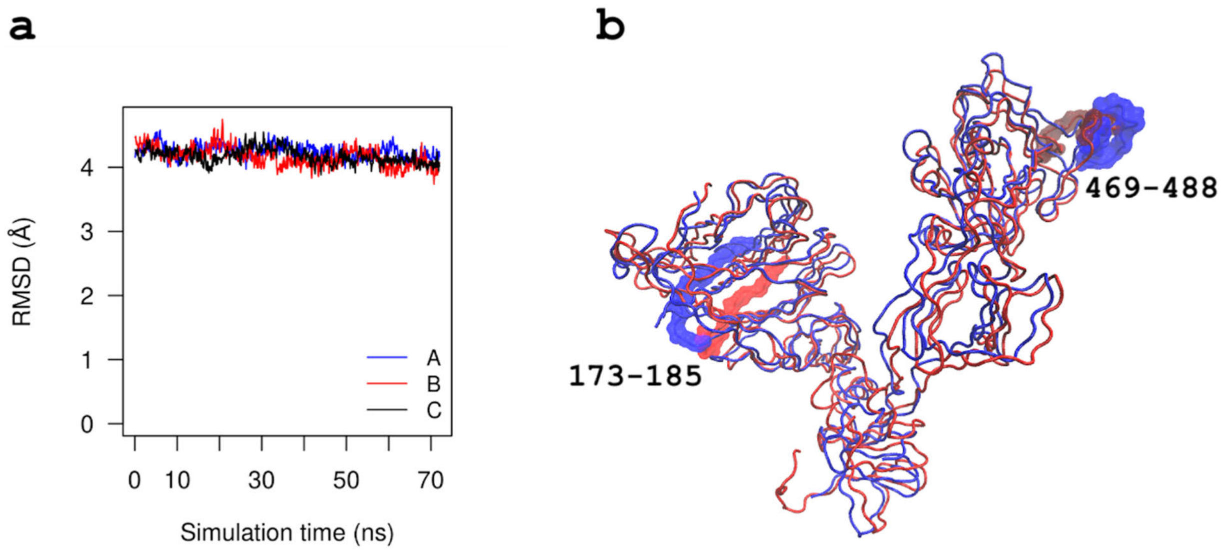

Figure S12. *Model comparison. (a) the time evolution of RMSD of alpha carbons in the model 6VXX_1_1_1 from cryo-EM structure 6ZGE in the last 70 ns of equilibration. A, B, and C represent chain A, chain B, and chain C. (b) The RMSD mainly comes from ill-matched two segments: residue id 173 to 185 and residue id 469 to 488. 6ZGE is colored blue. 6VXX_1_1_1 is colored red.*
